# Targeting Redox Imbalance as an Approach for Diabetic Kidney Disease

**DOI:** 10.3390/biomedicines8020040

**Published:** 2020-02-22

**Authors:** Keiichiro Matoba, Yusuke Takeda, Yosuke Nagai, Tamotsu Yokota, Kazunori Utsunomiya, Rimei Nishimura

**Affiliations:** 1 Division of Diabetes, Metabolism, and Endocrinology, Department of Internal Medicine, The Jikei University School of Medicine, Tokyo 105-8461, Japan; ms05-takeda@jikei.ac.jp (Y.T.); y.nagai@jikei.ac.jp (Y.N.); yokotat@jikei.ac.jp (T.Y.); rimei@jikei.ac.jp (R.N.); 2 Center for Preventive Medicine, The Jikei University School of Medicine, Tokyo 105-8461, Japan; kazu-utsunomiya@jikei.ac.jp

**Keywords:** diabetic kidney disease, oxidative stress, redox imbalance

## Abstract

Diabetic kidney disease (DKD) is a worldwide public health problem. It is the leading cause of end-stage renal disease and is associated with increased mortality from cardiovascular complications. The tight interactions between redox imbalance and the development of DKD are becoming increasingly evident. Numerous cascades, including the polyol and hexosamine pathways have been implicated in the oxidative stress of diabetes patients. However, the precise molecular mechanism by which oxidative stress affects the progression of DKD remains to be elucidated. Given the limited therapeutic options for DKD, it is essential to understand how oxidants and antioxidants are controlled in diabetes and how oxidative stress impacts the progression of renal damage. This review aims to provide an overview of the current status of knowledge regarding the pathological roles of oxidative stress in DKD. Finally, we summarize recent therapeutic approaches to preventing DKD with a focus on the anti-oxidative effects of newly developed anti-hyperglycemic agents.

## 1. Introduction

The current diabetes pandemic has emerged as a global health burden. Despite accumulating evidence supporting the prevention of obesity and related metabolic disorders, the number of diabetic patients is rapidly increasing, particularly in middle- and low-income countries [1]. According to the estimates of the International Diabetes Federation, diabetes affects 425 million people globally, and the number is expected to increase to more than 600 million in 2045 [2].

It is a major concern that diabetes is associated with the development of micro and macrovascular complications. Diabetic kidney disease (DKD) is the leading cause of end-stage renal disease and is therefore a critical issue for healthcare systems [3,4,5]. The reason for the diabetes pandemic can be explained, at least in part, by the aging of the population and the increase in obese subjects. DKD is characterized by functional and structural abnormalities in the glomeruli, including glomerular hyperfiltration, mesangial expansion, the thickening of the glomerular basement membrane, and podocyte loss. These changes ultimately result in the glomerulosclerosis associated with albuminuria and a decline in the glomerular filtration rate (GFR). Numerous studies have assessed the link between albuminuria and the cardiovascular risk in patients with DKD and have demonstrated that albuminuria is not only a hallmark of DKD, but also an independent risk factor for cardiovascular death. The detrimental interaction between diabetic renal injury and coronary heart disease is termed reno-cardiac syndrome [6]. Indeed, in the UK Prospective Diabetes Study (UKPDS), annual cardiovascular mortality rates were found to increase to 3%, 4.6%, and 19.2% with the progression to microalbuminuria, macroalbuminuria, and renal failure, respectively [7]. Importantly, cardiovascular events were the most common cause of death in all stages of DKD. It is therefore essential to establish effective therapeutic strategies against DKD. To this end, a detailed understanding of the molecular basis that drives DKD is required.

Emerging evidence suggests that DKD is associated with multiple metabolic disorders (e.g., hyperglycemia, hypertension, and dyslipidemia), hemodynamic changes, activation of the renin-angiotensin system (RAS), and inflammation [8]. While there is no doubt that multifactorial intervention for these abnormalities is necessary for the prevention of DKD acceleration [9,10], the current standards of care do not eliminate the risk of DKD. Given the limited therapeutic options for inhibiting DKD, there has been an ongoing effort to elucidate the biological mechanisms responsible for renal injury and to develop novel drugs.

There is a growing appreciation of the roles of oxidative cellular injury caused by free radicals in the process of diabetic vascular complications. Oxidative stress is defined as an imbalance between the formation of highly reactive molecules and the antioxidant mechanism. Reactive oxygen species (ROS) are chemicals, such as superoxide anion (O_2_-), hydroxyl radical (OH), and hydrogen peroxide (H_2_O_2_), which are generated in cells. Nicotinamide adenine dinucleotide phosphate (NADPH) oxidase is the most important source of superoxide anion and is upregulated in the kidney in diabetes [11]. Consistent with this data, superoxide is increased in the diabetic kidneys and in renal cells incubated under high glucose conditions. Moreover, excess free fatty acids, mainly derived from the obese state, induce the generation of ROS through oxidative phosphorylation in mitochondria. Another possible reason for the oxidative stress in diabetes is decreased antioxidants. As a result, excess ROS can damage DNA, protein, and lipids, making them nonfunctional. These findings highlight the importance of oxidative stress in the mechanisms that facilitate renal injury in the context of diabetes. We herein review the roles of oxidative stress in diabetic renal damage and finally describe medicines that have the potential to inhibit the renal ROS production in patients with DKD.

## 2. Oxidative Stress in Diabetes and DKD

**Figure 1:**
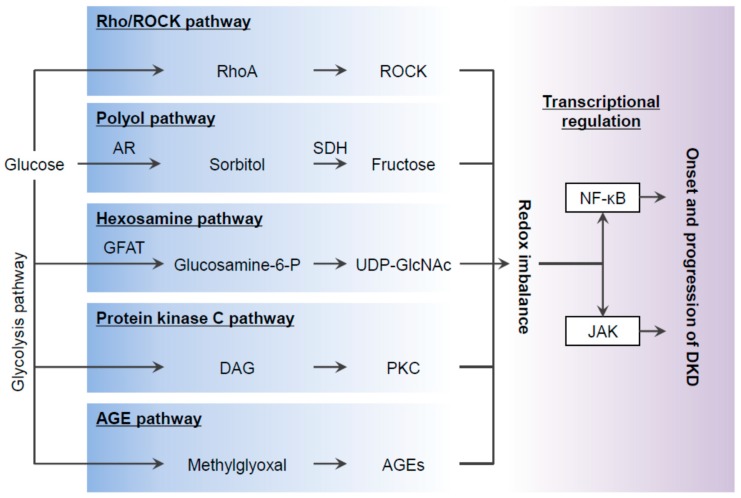
In the setting of diabetes, excess glucose activates several pathways including the polyol pathway, hexosamine pathway, as well as signals that lead to activation of Rho-kinase (ROCK), PKC and generation of AGEs. All of these activation results in oxidant/antioxidant imbalance that proceeds to DKD. AR, aldose reductase; SDH, sorbitol dehydrogenase; GFAT, glutamine:fructose-6-phosphate aminotransferase; glucosamine-6-P, glucosamine-6-phosphate; UDP-GlcNAc, uridine diphosphate-N-acetyl glucosamine; DAG, diacylglycerol; PKC, protein kinase C; AGEs, advanced glycation end-products.

Elevated ROS is considered to be a causal link between high glucose conditions and DKD progression. The various possible sources for the overproduction of ROS in diabetes include (Figure 1): (1) Activation of the polyol pathway leading to the accumulation of sorbitol and fructose, NADPH redox imbalances, and changes in signal transduction; (2) The increased flux of the hexosamine pathway; (3) Increased protein kinase C (PKC) activity and subsequent cascades of stress; (4) The non-enzymatic glycation of proteins generating advanced glycation end-products (AGEs); and (5) Activation of the small GTPase Rho and its target, Rho-kinase (ROCK).

### 2.1. The Polyol Pathway

Among the various pathways, the polyol pathway plays an important role in the development of DKD. It has been suggested that the polyol pathway generates not only osmotic stress, but also hyperglycemic oxidative stress in renal tissue. Under homeostatic conditions, cellular glucose is predominantly oxidized into glucose-6-phosphate and enters the glycolytic pathway to generate energy in the form of ATP. In states such as diabetes, however, the flux of glucose through the polyol pathway is increased.

In the polyol pathway, glucose is converted to sorbitol by aldose reductase (AR), the first and rate-limiting enzyme of this pathway, with the aid of its cofactor NADPH. AR has been detected in a number of tissues, including tissue of the liver, skeletal muscle, heart, eyes, neuron, and kidney. It has been extensively studied for a potential role in diabetic vascular complications including DKD [12]. In physiological settings, AR breaks down the toxic aldehyde products produced by lipid peroxidation, such as 4-hydroxyl-nonenal (HNE) and its glutathione conjugates (GSH-NHE) into inactive alcohols. Whereas the catalytic action of AR to mediate glucose reduction is negligible under euglycemic conditions, this enzyme is activated in diabetes. Not only in diabetic rodents, but the activity of AR is also increased in renal glomeruli in people with diabetes [13]. Supporting these findings, the accumulation of sorbitol has been demonstrated in glomerular mesangial cells incubated under high glucose conditions [14], which can induce cellular osmotic stress and damage diabetic glomeruli [15]. The fact that AR inhibitor treatment can prevent the development of DKD highlights the significance of the polyol pathway in the biology of DKD. For example, epalrestat, an inhibitor of AR, can attenuate albuminuria, podocyte foot process fusion, and interstitial fibrosis in type 2 diabetic db/db mice [16]. Moreover, AR-deficient mice are resistant to the progression of diabetic kidney injury [17]. In these mice, glomerular hypertrophy, extracellular matrix accumulation, and the overproduction of collagen 4 were inhibited in comparison to wild type mice. The increase in urinary albumin excretion was also prevented in the AR-null mice. In contrast to this experimental evidence, AR inhibitors only have a partial effect in preventing DKD in patients [18]. The incongruity between animal and human data may be due to the short length of clinical trials and variable potency of AR inhibitors.

As the second step in the polyol pathway, sorbitol is oxidized to fructose by sorbitol dehydrogenase (SDH), with its co-factor nicotinamide adenine dinucleotide (NAD). As such, the increased polyol flux leads to an increase in the NADH/NAD ratio. Since NAD is an important factor in many enzyme-catalyzed reactions, other metabolic pathways are also affected. The sirtuin signaling pathway is a major pathway affected by the reduction of NAD. Mammalian sirtuin is a NAD-dependent protein deacetylase with various functions in energy homeostasis, cell survival, and DNA repair [19]. As a consequence, decreased NAD levels eventually lead to the over-acetylation of proteins. Thus, supplementation with NAD precursors or analogues can serve as an effective therapeutic intervention for recovering sirtuin activity and preventing diseases, including diabetes and DKD [20,21]. Moreover, an NADH/NAD redox imbalance can reduce the availability of NADPH for regenerating antioxidant GSH. Importantly, NADH is used as a substrate for NADH oxidase to generate superoxide anions and this occurs in diabetes. Moreover, excess NADH also inhibits glycolysis and the subsequent Krebs cycle, which provides more glucose in the polyol pathway.

### 2.2. The Hexosamine Pathway

It is now clear that the increased flux of fructose-6-phosphate (fructose-6-P) into the hexosamine pathway also contributes to oxidative stress in the context of diabetes. Under normoglycemic conditions, most of the glucose is metabolized through the glycolysis pathway, and only 2–5% enters the hexosamine pathway and a small amount of fructose-6-P is produced [22]. However, under hyperglycemic conditions, excess fructose-6-P is derived from this pathway to provide a substrate for glutamine:fructose-6-phosphate aminotransferase (GFAT), the rate limiting enzyme of the hexosamine pathway. GFAT mediates the conversion of fructose-6-P to glucosamine-6-phosphate (glucosamine-6-P), and the consequent uridine diphosphate-N-acetyl glucosamine (UDP-GlcNAc).

UDP-GlcNAc is used for the production of glycosyl chains of proteins and lipids. Specific cytoplasmic and nuclear proteins are modified post-translationally by UDP-GlcNAc, and over-modification leads to pathologic changes in the gene expression. For example, transcriptional factors, such as specificity protein 1 (SP1), are modulated by UDP-GlcNAc [23] which leads to the induction of transforming growth factor β1 (TGF-β1) and plasminogen activator inhibitor-1 (PAI-1). Both of these factors contribute to the accumulation of extracellular matrix and diabetic glomerular sclerosis [24]. Consistent with these data, it has been reported that the inhibition of GFAT attenuates the hyperglycemia-induced increases of TGF-β1 in mesangial cells [25]. UDP-GlcNAc also stimulates the production of ROS and pro-apoptotic caspase-3 activity, which contribute to mesangial cell damage [26].

Importantly, the overexpression of GFAT results in the elevation of nuclear factor κB (NF-κB) promotor activity in mesangial cells [27], and excess UDP-GlcNAc provokes oxidative renal cell damage by upregulating the expression of tumor necrosis factor α (TNF-α). Given the well-established link between inflammation and oxidative stress [28], it is reasonable to suggest that the excess glucose flux through the hexosamine pathway leads to redox imbalance, at least in part, via the inflammatory response. Taken together, the activated hexosamine pathway is a piece of one machinery that diabetes leads to oxidative renal damage. Although recent investigations have expanded our knowledge regarding the molecular mechanisms of the hexosamine pathway, its pathological role in patients remains to be established.

### 2.3. The PKC Pathway

In the setting of diabetes, the increased flux of dihydroxyacetone phosphate (DHAP) to glycerol-3-phosphate leads to the generation of diacylglycerol (DAG), a physiological activator of PKC. PKC phosphorylates subunits of NADPH oxidase, which has been recognized to contribute to the production of ROS. The denouement of elevated PKC signaling is the activation of MAPK signaling and inducible nitric oxide synthase, culminating in structural and functional derangements. Indeed, the PKC-mediated oxidant production elicits endothelial dysfunction and apoptosis. By blocking PKC, the metabolic signaling cascade is interrupted and the production of ROS is reduced. Inhibiting the formation of DAG is one of the options for regulating the PKC signaling pathway.

PKC consists of a family of at least 12 members. PKC-α, PKC-β, PKC-δ, PKC-ε, and PKC-ζ are detected in the glomeruli [29]. PKC plays a pivotal role in renal dysfunction under diabetic conditions. It has been demonstrated that PKC isoforms are activated in diabetic glomeruli and mesangial cells cultured in high glucose media, which contribute to mesangial expansion as well as thickening of the glomerular basement membrane. The importance of PKC-β in DKD has been demonstrated in a loss-of-function study in mice. The amelioration of histological abnormalities and TGF-β1 induction was achieved in PKC-β knockout mice [30]. The urinary excretion of 8-hydroxydeoxyguanosine (8-OHdG) and isoprostane—parameters of oxidative stress—in PKC-β-deficient diabetic mice were reduced in comparison to wild-type mice. Mechanistically, the activity of NADPH oxidase and the mRNA expression levels of p47phox, Nox2, and Nox4 were attenuated in these mice. These findings, coupled with the work of others, have led to the increasing appreciation that PKC-β is an essential determinant of the ROS production.

### 2.4. The AGE-RAGE Pathway

Another important pathway whereby glucose can activate oxidative stress is AGE signaling. AGEs are generated exogenously and endogenously through non-enzymatic reactions of amino acids, proteins, glucose, and metabolites (e.g., glycolaldehyde, glyceraldehyde, glyoxal, methylglyoxal, 3-deoxyglucosone, and fructose) [31]. The receptor for AGE (RAGE) is a multi-ligand cell surface receptor that can be induced under diabetic conditions. The AGE-RAGE system is one of the major pathways involved in the onset and progression of diabetic vascular complications, including neuropathy, retinopathy, and nephropathy. The flux of glucose through the polyol pathway would increase the formation of toxic AGE compounds, ultimately leading to the production of free radicals, and a decrease in nitric oxide and taurine. Therefore, there is crosstalk between AR-dependent and -independent mechanisms of oxidative stress, which makes it difficult to understand the relative contributions of each. AGEs are known to accumulate intracellularly, which stimulates various signaling pathways, including the inflammatory response. Accumulated AGEs specifically stimulate the production of inflammatory cytokines and adhesion molecules via regulation of transcriptional factor NF-κB, as well as the PKC pathway. The proposed mechanisms by which AGEs interfere with these functions include the disruption of molecular confirmation and the reduction of the degradation capacity by its deposition or activation of RAGE.

In renal mesangial cells, type 4 collagen is upregulated by exposure to AGEs. However, this induction was prevented by the anti-RAGE ribozyme, suggesting that the AGE-mediated induction of the expression of type 4 collagen is dependent on RAGE. It has indeed been reported that diabetic mice that overexpress human RAGE in vascular cells exhibited kidney enlargement, glomerular hypertrophy with mesangial expansion and increased urinary albumin excretion [32]. Moreover, these phenotypes were prevented by the administration of an AGE inhibitor, indicating that the AGE-RAGE signal is an effective target for overcoming DKD. Other studies have evaluated the effects of the specific inhibition of RAGE with DNA aptamer and demonstrated that RAGE-aptamer attenuates the development of experimental DKD by inhibiting oxidative stress [33,34].

### 2.5. ROCK Signaling

The small GTP-binding protein Rho and its downstream target Rho-associated coiled-coil containing protein kinase (Rho-kinase, ROCK) are implicated in a variety of cellular processes, including cell proliferation, contraction, and migration. ROCK signaling is activated by glucose, cytokines, and ROS [35,36,37]. Initial insights linking ROCK to diabetic vascular complications were gleaned from our studies identifying ROCK as a critical molecule of the pathological changes of the microvasculature and large blood vessels. For instance, the inhibition of ROCK results in the attenuation of albuminuria, glomerular hypertrophy, and macrophage infiltration in mouse models of diabetes [24,38]. Furthermore, the beneficial effects of ROCK inhibition are observed in the diabetic retina and neuron. Yokota et al. showed that enhanced expression of retinal vascular endothelial growth factor (VEGF) was attenuated by the treatment with ROCK inhibitor in streptozotocin-induced diabetic rat [39]. ROCK blockade also restores normal motor nerve conduction velocity in diabetic rats by mediating proper localization of adhesion-related molecules in myelinating Schwann cells [40]. Another study in endothelial cells demonstrated that ROCK acts as a key player in vascular inflammation [41].

ROCK has two isoforms (ROCK1 and ROCK2) that share 65% sequence homology, but have different activation machinery. Genetic deletion models of ROCK1 and ROCK2 revealed distinctive roles in the regulation of cellular function. For instance, ROCK1 knockout modes demonstrate impaired closure of eyelid and umbilical ring [42], whereas ROCK2 deletion leads to intrauterine growth retardation [43]. Takeda et al. showed the strong contribution of endothelial ROCK2 to the induction of adhesion molecules and chemokines via NF-κB activation [37]. In addition, Nagai et al. reported ROCK2-mediated fibrotic reactions in mesangial cells [36]. Taken together, these findings raise the possibility that ROCK isoform-specific therapeutic approach may be effective for the prevention of diabetic vascular complications.

We provided evidence implicating ROCK as an essential regulator of the redox balance and the progression of DKD [44]. In the streptozotocin-induced diabetic rat, renal Nox4 (a catalytic subunit of NADPH oxidase) and urinary 8-OHdG were increased, suggesting oxidative stress in the diabetic kidney. Of note, ROCK inhibitor attenuated the renal Nox4 expression and the urinary increase of 8-OHdG. Moreover, ROCK regulates the mesangial production of inflammatory cytokines through the mechanism of NF-κB [45]. Whereas some experiments have identified the direct regulation of ROCK in either IκBα degradation or p65 phosphorylation, a mechanism through which ROCK is involved in the nuclear import of p65, without affecting either IκBα degradation or the phosphorylation of p65, has also been documented [38]. Collectively, these findings suggest a direct or inflammation-mediated effect of ROCK on oxidative stress in the diabetic kidney.

## 3. ROS-Mediated Stress Signaling in DKD

As described above, ROS is produced under diabetic conditions. In addition to its direct effects on cellular protein and DNA, ROS activates several signaling cascades that are implicated in the histological and functional changes in DKD.

### 3.1. NF-κB and AP-1

Experimental work over the past decade has revealed the mechanistic basis of the interplay between inflammation and oxidative stress. The inflammatory signal is mainly orchestrated by transcriptional factor NF-κB, which consists of a heterodimer of the Rel family proteins: p65 and p50. Under physiological conditions, NF-κB is sequestered in the cytoplasm by IκB family proteins, the best characterized of which is IκBα. The phosphorylation of IκBα results in its ubiquitination and subsequent proteasomal degradation, which results in the exposure of its nuclear localization signal. The following recognition of NF-κB by karyopherin β directs it to the nuclear pore complex, thus allowing for entry into the nucleus. The coactivator p300 generates a complex with p65/p50 dimer to stabilize the chromatin structure for efficient transcription.

Increased renal NF-κB levels are detected in the kidneys of diabetic experimental models, which activate glomerular and tubular cells to induce renal injury [35,45]. Downstream targets of NF-κB include adhesion molecules and pro-inflammatory cytokines (e.g., interleukin 6, TNF-α), which all drive oxidative stress and the development of DKD. In addition to the role as downstream events of NF-κB activation, ROS activates NF-κB signaling [46]. For instance, H_2_O_2_ stimulates NF-κB to produce an array of inflammatory mediators. In turn, these activate the further production of ROS. Activating protein 1 (AP-1) is another major redox-sensitive transcriptional factor. ROS activates c-Fos and c-Jun, both of which are components of AP-1. Similar to NF-κB, glucose and oxidative stress activate AP-1-mediated inflammatory signaling, which will contribute to the formation of the vicious inflammation-oxidative stress cycle. Further clinical trial and mechanistic investigations are required in order to validate the role of AP-1 in the pathogenesis of DKD.

### 3.2. JAK-STAT

Janus kinase/signal transducer and activator of transcription (JAK-STAT) is activated by extracellular ligands, such as cytokines, chemokines, and growth factors to induce a number of cellular responses. ROS has also been shown to activate the JAK-STAT pathway under excessive glucose levels and is associated with glomerular expansion [47]. For example, H_2_O_2_ stimulates the activity of JAK family members, JAK2 and tyrosine kinase 2 (TYK2) [48].

The fundamental role of the JAK-STAT signal is originally established in lymphoid cells. However, this signal also has definitive roles in renal cells (i.e., glomerular mesangial cells, podocytes, and tubular epithelial cells) [49]. The overexpression of podocyte JAK2 results in the worsening of renal injury in mouse models of DKD [50]. As a bridge to clinical translation, the activation of JAK-STAT signaling is observed in the kidney of DKD patients. A transcriptomic investigation performed in glomerular samples obtained from subjects with DKD has shown that all downstream targets of JAK-STAT were highly expressed in the glomeruli of patients in comparison to healthy subjects. Notably, the degree of gene induction of the JAK-STAT pathway was tightly and inversely correlated with the decline in GFR. Moreover, Baricitinib, an inhibitor of the JAK family of protein tyrosine kinases that selectively inhibits JAK1 and JAK2, attenuates albuminuria in diabetic patients. As such, inhibiting oxidative stress may have the potential to block JAK-STAT signaling and the progression of DKD.

### 3.3. Nrf2-Keap1

The antioxidant defense mechanism (e.g., superoxide dismutase, glutathione reductase, glutathione peroxidase, and GSH) is essential for scavenging of ROS. However, this system is impaired in diabetes [51]. The defense response is mainly regulated by nuclear factor erythroid 2-related factor 2 (Nrf2), which governs the expression of antioxidants and detoxification enzymes. The cytosolic inhibitor Kelch-like ECH-associated protein 1 (Keap1) acts as a “sensor” for cellular oxidative stress. Keap1 is proposed to mediate Nrf2 activity via its capacity to inhibit Nrf2 nuclear translocation.

The upregulation of Nrf2-Keap1-dependent antioxidants attenuates renal oxidative overload. Notably, activation of Nrf2 improves histological findings in the glomerulus of streptozotocin-induced diabetic mice by attenuating oxidative stress, the expression of TGF-β1, and the production of extracellular matrix [52]. In addition, Nrf2 inhibits glucose-induced mesangial hypertrophy. Although the Nrf2 activator known as bardoxolone has been demonstrated to be reno-protective in patients with DKD, significant increase of heart failure was reported in the bardoxolone–treated group [53,54,55]. A clinical trial excluding high-risk patients is ongoing in Japan [56]. Moreover, bardoxolone is currently being studied in patients with Alport syndrome enrolled in the United States, Europe, Japan, and Australia. The potential to prevent or delay kidney function decline will be evaluated in these studies.

## 4. Targeting Oxidative Stress for the Prevention of DKD

Therapeutic options for the management of DKD are currently limited to systemic intervention for diabetes-related metabolic changes (i.e., hyperglycemia, hypertension, and dyslipidemia). Given the importance of oxidative stress for many of the pathological aspects of kidney injury, the redox imbalance could be a potential therapeutic target for the prevention of renal failure in diabetic patients. Several studies have shown that the inhibition of oxidative stress can prevent metabolic dysfunction, fibrotic signals, and proteinuria. Agents that are already in use in clinical practice such as statins, metformin, and thiazolidinediones have been demonstrated to suppress oxidative stress by inhibiting NADPH oxidase [57,58]. In addition to these drugs, recent clinical trials have established compelling evidence of beneficial cardiorenal outcomes of sodium glucose co-transporter 2 (SGLT2) inhibitors and glucagon-like peptide-1 (GLP-1) receptor agonists, beyond their glucose-lowering effect. The regulation of oxidative stress is one of the assumed mechanisms of the beneficial effects of these agents.

### 4.1. SGLT2 Inhibitors

SGLT2 inhibitors are a novel class of anti-hyperglycemic agents that are being used increasingly frequently for the management of diabetes. This drug blocks SGLT2 at the renal proximal tubule, leading to the limitation of glucose reabsorption of filtered glucose, which in turn results in glycosuria and blood glucose reduction. This effect is independent of the action of insulin. In addition to its glucose-lowering action, SGLT2 inhibitors induce natriuresis, thereby causing a reduction of blood pressure, improvement of glomerular hyperfiltration and albuminuria, at least in part, by activating tubuloglomerular feedback [59].

The Food and Drug Administration mandate the assessment of the cardiovascular safety of all novel hypoglycemic agents prior to seeking approval. The secondary outcome analyses in these trials have demonstrated the potential of SGLT2 inhibitors to reduce the risks of DKD and end-stage renal disease. Empagliflozin has been shown to improve renal composite outcomes (i.e., doubling of creatinine, renal replacement therapy, or renal death) in patients with type 2 diabetes [60]. Similar results were reported in trials with canagliflozin. The Canagliflozin and Renal Endpoints in Diabetes with Established Nephropathy Clinical Evaluation (CREDENCE) trial was conducted to investigate the efficacy and safety of canagliflozin for attenuating clinically important cardiorenal outcomes in patients with DKD [61]. Importantly, the CREDENCE trial was restricted to patients who were already taking the maximum tolerated dose of an angiotensin-converting enzyme inhibitor or angiotensin receptor blocker. A significant reduction was observed in the relative risk of the renal composite outcomes in subjects treated with canagliflozin. In addition to the renal benefits, the rates of cardiovascular death, myocardial infarction, or stroke and hospitalization for heart failure were lower in the canagliflozin group. Remarkably, there was no increase in the rates of amputation or bone fracture. Based on these large clinical trials, SGLT2 inhibitors are recommended for high-risk DKD patients with an estimated GFR of >30 mL/min/1.73 m^2^ or urinary albumin excretion >30 mg/g creatinine (particularly >300 mg/g creatinine), to reduce the risk of DKD progression [3,62].

Decreased GSH levels and elevated oxidized glutathione (GSSG) levels are detected in the renal cortex of type 2 diabetic mice. Tanaka et al. showed that ipragliflozin treatment ameliorated albuminuria, glomerular expansion with the improvement of this redox imbalance [63]. The administration of empagliflozin also attenuated renal fibrosis, inflammation, as well as elevation of 8-OHdG in streptozotocin-induced diabetic mice [64]. Furthermore, the glucose-induced ROS generation was suppressed by siRNA against SGLT2 in tubular cells [65].

The basic mechanism to explain the effects on oxidative stress may involve anti-inflammatory actions of SGLT2 inhibitors [59]. Diabetic patients treated with canagliflozin showed lower plasma levels of TNF receptor and interleukin 6 in comparison to glimepiride group [66]. Animal studies also suggested anti-inflammatory actions of SGLT2 inhibitors. For example, empagliflozin significantly reduced mRNA levels of inflammatory mediators, such as monocyte chemoattractant protein 1 and interleukin 6 [67]. Additionally, SGLT2 inhibitors have been shown to modulate energy metabolism by inducing energy loss, fat utilization, and the browning of white adipose tissue [68]. Therefore, the beneficial effects of SGLT2 inhibitors on renal oxidative stress are partly mediated by weight loss, the improvement of insulin resistance, and the reduction of adipose tissue inflammation.

### 4.2. GLP-1 Receptor Agonists

GLP-1 is a gut-derived hormone secreted from intestinal L-cells upon meal ingestion that was originally found to mediate glucose handling by regulating the pancreatic islet cell activity, food intake, and gastrointestinal function. In addition to pancreatic β cells, the GLP-1 receptor is detected in renal tubular cells and the kidney vessels. Activation of GLP-1 receptor stimulates adenylate cyclase to drive the production of cAMP and protein kinase A (PKA) activation, primary effectors of GLP-1-mediated insulin secretion. Findings from small clinical studies as well as large cardiovascular safety trials with GLP-1 receptor agonists suggest its renoprotective effects, mainly in patients with macroalbuminuria [69,70,71]. However, no change was observed in renal hard endpoints, possibly due to low the incidence of renal death. It is reasonable to suggest that the beneficial actions of GLP-1 receptor agonists are partly through effects on body weight and associated risk factors for DKD. Similarly to SGLT2 inhibitors, natriuresis is induced by the GLP-1-mediated inhibition of the sodium-hydrogen exchanger 3, which is located at the brush border of the renal proximal tubule. In addition, GLP-1 receptor agonists improve dyslipidemia by reducing the production and secretion of intestinal chylomicrons. However, given the finding that beneficial effects of GLP-1 receptor agonists on albuminuria were observed even after adjustment for HbA1c and other traditional metabolic abnormalities, it is plausible that direct actions contributed to their effects on albuminuria.

Studies have shown that cAMP and PKA signaling are linked to the anti-oxidative response. GLP-1-mediated cAMP elevation demonstrates anti-oxidative effects by inhibiting AGE-RAGE signaling [72]. Therefore, it is likely that GLP-1 receptor agonists are protective against renal oxidative stress, independently of blood glucose levels. Indeed, disruption of the GLP-1 receptor in mice resulted in the progression of DKD with the elevation of renal NADPH and superoxide. Conversely, liraglutide suppressed these inductions through the actions on cAMP and PKA activity [73]. Of note, these improvements have been observed without major alterations in glucose metabolism, further supporting the hypothesis of direct renal effects of GLP-1 receptor agonists.

## 5. Conclusions and Future Perspectives

Given the high mortality of DKD and increased healthcare costs, kidney protection is a critical issue in the management of diabetes. There is now satisfactory evidence to illustrate the causal link between oxidative stress and the progression of DKD. When considered alongside the fact that it is still difficult to diagnose at an early stage due to a lack of reliable biomarkers, serum or urinary oxidative stress markers may be useful for making a diagnosis of DKD, especially with the combination of urinary albumin levels. Whether redox imbalance is either a trigger or consequence of DKD progression is still debated. However, it is conceivable that it is both. Experimental studies using gene deletion models of specific enzymes or kinases have greatly expanded our understanding of oxidative stress. In addition to traditional antioxidants such as vitamin C and E, clinical and experimental data support the possibility that novel classes of anti-hyperglycemic agents have beneficial effects on oxidative stress and the renal outcome. In particular, SGLT2 inhibitors and GLP-1 receptor agonists are potential pharmaceuticals that could reduce renal oxidative stress through direct or indirect actions. A robust understanding of the mechanism underlying these agents’ renal benefits will be required to facilitate the establishment of novel therapeutic strategies against DKD.
